# BOHEMIA: Broad One Health Endectocide-based Malaria Intervention in Africa—a phase III cluster-randomized, open-label, clinical trial to study the safety and efficacy of ivermectin mass drug administration to reduce malaria transmission in two African settings

**DOI:** 10.1186/s13063-023-07098-2

**Published:** 2023-02-21

**Authors:** Carlos Chaccour, Aina Casellas, Felix Hammann, Paula Ruiz-Castillo, Patricia Nicolas, Julia Montaña, Mary Mael, Prashant Selvaraj, Urs Duthaler, Sigilbert Mrema, Mwaka Kakolwa, Issa Lyimo, Fredros Okumu, Achla Marathe, Roger Schürch, Eldo Elobolobo, Charfudin Sacoor, Francisco Saute, Kang Xia, Caroline Jones, Cassidy Rist, Marta Maia, N. Regina Rabinovich

**Affiliations:** 1 ISGlobal, Barcelona Institute for Global Health, Barcelona, Spain; 2 Universidda de Navarra, Pamplona, Spain; 3 Centro de Investigación Biomédica en Red de Enfermedades Infecciosas, Madrid, Spain; 4 University Hospital of Bern, Inselspital, Bern, Switzerland; 5 Bill and Melinda Gates Foundation, Seattle, USA; 6 University Basel, Basel, Switzerland; 7 Ifakara Health Institute, Ifakara, Tanzania; 8 University of Virginia, Charlottesville, USA; 9 Virginia Polytechnic Institute and State University, Blacksburg, USA; 10 Centro de Investigação em Saúde de Manhiça, Manhica, Mozambique; 11 KEMRI Wellcome Trust Research Programme, Kilifi, Kenya; 12 TH Chan Harvard School of Public Health, Boston, USA

**Keywords:** Malaria, Ivermectin, Endectocides, Cluster-randomized, Mozambique

## Abstract

**Background:**

Residual malaria transmission is the result of adaptive mosquito behavior that allows malaria vectors to thrive and sustain transmission in the presence of good access to bed nets or insecticide residual spraying. These behaviors include crepuscular and outdoor feeding as well as intermittent feeding upon livestock. Ivermectin is a broadly used antiparasitic drug that kills mosquitoes feeding on a treated subject for a dose-dependent period. Mass drug administration with ivermectin has been proposed as a complementary strategy to reduce malaria transmission.

**Methods:**

A cluster randomized, parallel arm, superiority trial conducted in two settings with distinct eco-epidemiological conditions in East and Southern Africa. There will be three groups: human intervention, consisting of a dose of ivermectin (400 mcg/kg) administered monthly for 3 months to all the eligible population in the cluster (>15 kg, non-pregnant and no medical contraindication); human and livestock intervention, consisting human treatment as above plus treatment of livestock in the area with a single dose of injectable ivermectin (200 mcg/kg) monthly for 3 months; and controls, consisting of a dose of albendazole (400 mg) monthly for 3 months. The main outcome measure will be malaria incidence in a cohort of children under five living in the core of each cluster followed prospectively with monthly RDTs

**Discussion:**

The second site for the implementation of this protocol has changed from Tanzania to Kenya. This summary presents the Mozambique-specific protocol while the updated master protocol and the adapted Kenya-specific protocol undergo national approval in Kenya. BOHEMIA will be the first large-scale trial evaluating the impact of ivermectin-only mass drug administration to humans or humans and cattle on local malaria transmission

**Trial registration:**

ClinicalTrials.govNCT04966702. Registered on July 19, 2021.

Pan African Clinical Trials Registry PACTR202106695877303.

## Administrative information

Note: the numbers in curly brackets in this protocol refer to SPIRIT checklist item numbers. The order of the items has been modified to group similar items (see http://www.equator-network.org/reporting-guidelines/spirit-2013-statement-defining-standard-protocol-items-for-clinical-trials/).

**Table:** 

Title {1}	BOHEMIA: Broad One Health Endectocide-based Malaria Intervention in Africa—a phase III cluster-randomized, open-label, clinical trial to study the safety and efficacy of ivermectin mass drug administration to reduce malaria transmission in two African settings
Trial registration {2a and 2b}.	PACTR: PACTR202106695877303 ClinicalTrials.gov: NCT04966702
Protocol version {3}	4.2, dated 13/march/2022
Funding {4}	Unitaid
Author details {5a}	ISGlobal, Barcelona Institute for Global Health: CCh, AC, PRC, PN, JM, MM, RR Universidad de Navarra: CCh CIBERINFEC: CCh University Hospital Bern: FH Bill and Melinda Gates Foundation: PS University of Bassel: UD Ifakara Health Institute: SM; MK; IL; FO University of Virginia: AM Virginia Tech: RS, KX, CR Centro de Investigacao em Saude de Manhica: EE, CS, FS Kemri-Wellcome research programme: CJ, MMaia TH Chan Harvard School of Public Health: RR
Name and contact information for the trial sponsor {5b}	ISGlobal - Barcelona Institute for Global Health; Carrer Roselló 132, 5° 2ª, 08036, Barcelona, Spain
Role of sponsor {5c}	The sponsor designed the study, and participated in analysis and interpretation the data. Publication decisions are guided by consortium-wide publication policy and plan.

## Introduction

### Background and rationale {6a}

This protocol was submitted to *Trials* after recruitment completion but before the last patient/last visit expected in March 2023. This is because full approval was only obtained the day before implementation and the first months of the study coincided with floods caused by the Gombe cyclone. This followed a period of intense fieldwork in the context of a cholera outbreak while simultaneously managing the change to an alternative site for the second study in Kenya given the impossibility to work in Tanzania due to political rumors in the selected district. As of the submission of this protocol, data collection is ongoing and the analysis team remains blinded to group assignment.

Malaria is preventable and treatable, and yet it remains a significant public health problem around the world. In 2020, there were 241 million cases and 627,000 malaria deaths globally. Around 93% of the cases and 94% of the deaths occurred in the WHO Africa Region [1]. Malaria disproportionally affects the livelihood of the rural poor and has a deep economic impact, since it both thrives in and perpetuates poverty [2].

Based on substantive progress in the 2000–2015 period, the World Health Organization (WHO), through the Global Technical Strategy (GTS) [3], proposed ambitious goals for malaria by 2030. These include the reduction of malaria cases by 90% as compared with the 2015 numbers and elimination in at least 35 countries. Achieving these goals could have a cumulative additional economic output of US$ 4.1 trillion [2].

However, the global fight against malaria is at a crossroads [4]. The 2017–2019 World Malaria Reports show that the decrease in cases and deaths had stalled, and the global program was off track to reach the 2030 milestones of the GTS. New and improved ways to fight malaria, particularly in the countries with the highest burden, are needed to get back on track. Moreover, the COVID-19 pandemic threatens to disrupt control programs and set back malaria to the levels of 20 years ago [5].

One critical challenge to achieving the 2030 goals is residual transmission, which is defined as “persistence of malaria transmission following the implementation in time and space of a widely effective malaria programme” [6]. This is driven by mosquito behavioral adaptations that allow transmission to continue even in presence of good coverage with core vector control tools such as long-lasting insecticidal nets (LLINs) and indoor residual spraying (IRS). Feeding outdoors, feeding early in the evening before bedtime, or leaving houses quickly after feeding, without resting indoors, are all behaviors that allow mosquitoes to avoid home-centered insecticides [7]. Additionally, temporarily feeding on livestock can allow mosquitoes to thrive and feed opportunistically on humans when available, contributing to sustained transmission [8, 9].

Endectocides are drugs that kill endo- and ectoparasites. Ivermectin is an endectocide licensed for human use in the 1980s. It has been a key drug in the treatment and control of onchocerciasis and lymphatic filariasis (LF) [10]. More than 3.7 billion treatments have been donated by Merck and mass-distributed at the population level over the last 30 years [11]. Ivermectin also kills mosquitoes that feed on treated humans or animals. This has led to the suggestion that mass drug administration (MDA) with ivermectin can contribute to killing mosquitoes regardless of their behavior and location, and thus to address residual transmission [12].

The capability of ivermectin to kill mosquitoes has been evaluated in dozens of insectary-based experiments of colony or wild mosquito survival after feeding on ivermectin-containing blood [13–39]. Although a variety of methods, doses, hosts and vector species have been evaluated, common results are:

- Ivermectin increases the mortality of *Anopheles* mosquitoes that ingest it in a blood meal.
- Mosquito mortality is directly related to the ivermectin-blood concentration (i.e., dose-response relationship).
- The lethal effect is driven by the time the drug is present in the blood and the concentration reached (area under the curve, AUC); the time-in-blood above a certain threshold is the most important factor.
- The drug causes a series of sublethal effects that can contribute beyond the killing effect to reduce transmission (mosquito knock-down, reduced fertility, reduce motility).
- It appears that different mosquito species have different degrees of susceptibility to the drug, some species such as *Anopheles arabiensis* or *Anopheles minimus* die quickly after imbibing low concentrations, while others such as *Anopheles dirus* or *Anopheles darlingi* can tolerate higher concentrations in their bloodmeal.

Additional evidence has emerged from mosquito collections conducted in onchocerciasis-endemic areas before and after ivermectin MDA was administered at the onchocerciasis dose of 150–200 mcg/kg [22, 23, 29]. These mosquito studies have shown three major effects:

- A reduction of 33% in the 3-day mosquito survival lasting for about 1 week after MDA,
- A shifting of the mosquito age population structure towards younger, less infectious, ages lasting for about 3 weeks, and
- A reduction of 77% in the proportion of mosquitoes carrying malaria parasites in their salivary glands (the sporozoite rate) lasting for about 2 weeks after MDA.

There are two published cluster-randomized trials of ivermectin MDA for malaria to date. One conducted in Burkina Faso concluded that six doses of ivermectin (150–200 mcg/kg) given three weeks apart reduced malaria incidence by 20% in children under five years of age who did not receive ivermectin, reflecting decreased transmission [40]. The other one, conducted in The Gambia, used ivermectin in combination with mass-drug administration of an antimalarial and found a decrease in transmission albeit the attributable fraction to each drug is not readily interpreted given the trial design.

Several modeling exercises have also assessed the potential impact of ivermectin MDA on malaria transmission [36, 41, 42] and concluded that it can reduce malaria transmission in different settings, with the critical variables being:

- The duration of the drug in the blood of treated subjects, reflecting both the dose and the regimen used,
- The coverage achieved in both human and livestock blood, and
- The timing of the intervention in relation to the transmission season.

Importantly, modeling suggests that adding ivermectin to other drug-based interventions, such as seasonal malaria chemoprevention (SMC) or MDA with antimalarials, could significantly enhance the effect and potentially reduce the number of rounds or coverage needed to achieve impact with the strategy.

BOHEMIA will conduct two cluster-randomized, individually powered trials in parallel in different eco-epidemiological settings [43]. The goal of these trials is to generate solid evidence to support the evaluation of ivermectin as a complementary vector control strategy for malaria prevention [44, 45].

The BOHEMIA trial will be carried out in Mopeia, Mozambique [46], and Kwale, Kenya. The primary research question is

*Does ivermectin MDA to the eligible human population in three monthly doses of 400 mcg/kg at the start of the rainy season (with or without including livestock) result in a relevant reduction of malaria transmission (as shown by a 20% reduction in infection incidence and supported by entomological measurements) with an acceptable safety profile?*

### Objectives {7}

Primary objective:

To determine the safety (in humans) and efficacy of ivermectin MDA (to humans or human and livestock simultaneously) for the prevention of malaria.

Note these co-primary objectives are determined in different populations. The efficacy endpoint is primarily measured in children under 5 years of age and safety is determined by anyone who receives the drug. Given the inclusion criteria, there might be a small overlap between the two populations.

Secondary objectives:

- To assess the efficacy of the intervention using complementary methods (efficacy)
- To assess the safety of the intervention with complementary methods (safety)
- To assess the PK of the proposed ivermectin dose/regime in its relationship with efficacy and safety outcomes (efficacy and safety)
- To assess the impact of ivermectin MDA at the proposed regimen on the prevalence of selected ectoparasitic NTDs (efficacy on NTDs and acceptability)
- To assess the relationship between malaria incidence in children and community prevalence at the peak of the malaria season (this serves for prevalence outcomes and paves the way for future studies or future evaluation using this outcome which could require fewer resources).
- To assess the relationship between active and passive surveillance for malaria at health facility (this serves as validation of passive surveillance and paves the way for future studies using this outcome which is much less resource-consuming while ensuring no key safety events are missed)
- To assess the accuracy for malaria diagnosis of two different malaria rapid diagnostic tests (RDTs) used in comparison to PCR (this is directly linked to efficacy as determined by RDTs)

### Trial design {8}

The BOHEMIA clinical trial has been designed in accordance with VCAG recommendations for conducting Phase III trials of traditional vector control tools in two different epidemiological settings [43]. The trial will use ivermectin as a first-in-class complementary vector control product to target residual transmission.

The clinical trial will be open-label, superiority, cluster randomized, controlled, parallel arm trial and will last one year. Given the nature of endectocides, which provide indirect protection through a community effect, the intervention must be randomized in clusters.

## Methods: participants, interventions, and outcomes

### Study setting {9}

The BOHEMIA cluster randomized trial will be carried out in Mozambique and Kenya. This summary presents the Mozambique-specific protocol while the updated master protocol and the adapted Kenya-specific protocol undergo national approval in Kenya.

### Eligibility criteria {10}

Inclusion criteria:

For human treatment/safety cohort

- Residents of the study area
- Male or female weighing more than 15kg
- Adult able to provide written consent
- Minors aged 12 to 17 able to provide assent
- Parent/guardian’s ability to provide consent for minors
- Negative pregnancy test for women aged between 13 and 49
- Agreement to adhere to study visits and procedures

For pediatric active cohort:

- Children in the age of highest burden at the time of enrollment (under 5 years of age)
- Residents of the study area
- Parent/guardian’s ability to provide consent for minors

For cross sectionals

- Residents of the area for at least 3 months prior to enrolment
- Parent/guardian’s consent for minors
- Ability to provide assent for minors aged 12 to 17
- Written consent from adults

For livestock treatment:

- Owner/guardian able to provide consent
- Animal expected to spend at least one week every study month inside the cluster border

Exclusion criteria:

For human treatment/safety cohort:

- Known hypersensitivity to ivermectin or albendazole
- Risk of Loa as assessed by travel history to Angola, Cameroon, Chad, Central African Republic, Congo, DR Congo, Equatorial Guinea, Ethiopia, Gabon, Nigeria, or Sudan
- Pregnant women
- Lactating women in the first week postpartum
- Children < 15 kg
- Currently participating in another clinical trial
- Unwilling to provide informed consent or assent
- Unwilling to adhere to study visits and/or procedures
- Severely ill either self-reported or in the eyes of the investigator, e.g., defined as the need for clinical care, or active or progressive disease interfering with activities of daily living. If in doubt, these criteria can be confirmed after a call with either the site PI/MD/safety officer against a pre-defined list.
- Currently under treatment with inhibitors of CYP3A or P-gp or other drugs that can interfere with the study

For active pediatric cohort:

- Non-residents
- Currently enrolled in other clinical trials

For cross sectionals

- Non-residents

For livestock treatment

- Received ivermectin less than 4 weeks ago
- Intention to milk or slaughter the animal for human consumption during the withdrawal period
- Calves under 8 weeks and piglets under 6 weeks of age.

### Who will take informed consent? {26a}

Appropriately trained and qualified field workers will visit the selected households and explain the study objectives, methods, and procedures to all household members who will be invited to participate in the study. The field workers will explain the inclusion and exclusion criteria and offer to answer questions as part of the informed consent process.

### Additional consent provisions for collection and use of participant data and biological specimens {26b}

Consent for shipping samples abroad is an explicit section of the ICF and participants can opt-out.

## Interventions

### Explanation for the choice of comparators {6b}

Participants in the control group will receive albendazole. The main rationale for this is ethical. While there is clear equipoise (that is, lack of evidence whether ivermectin works as intended) for the potential benefit of ivermectin regarding malaria transmission, ivermectin has proven efficacy against *Ascaris lumbricoides* and partial efficacy against *Trichuris trichura*, two of the most frequent STHs. Additionally, the effect of helminth infection on the human immune response is well established [47]; for malaria, there is limited evidence in this regard [48, 49]. Having a degree of deworming in both groups can help reduce confounding due to potential helminth-related immunoregulation.

Albendazole serves three purposes in the design of these trials:

- It provides participants in the control group with the benefit of deworming
- It helps with acceptability as participants in both groups will pass worms
- It increases comparability given some deworming in both groups

### Intervention description {11a}

#### Dosage and regimen

The ivermectin group will receive a single dose of 400 mcg/kg, given once a month for three months. The participant’s weight and related dosage will be confirmed using portable scales prior to administration.

The albendazole group will receive a single dose of 400 mg, given once a month for three months.

The following dosage forms will be used in the study:

- Ivermectin: 3 mg tablets
- Albendazole: 400 mg tablets
- Animal treatment: ivermectin injectable at 1% once a month

### Criteria for discontinuing or modifying allocated interventions {11b}

Participants experiencing drug-related severe adverse events will not be re-challenged

Women found pregnant will not receive further treatment.

### Strategies to improve adherence to interventions {11c}

The treatment will be administered under directly observed therapy on each round.

### Relevant concomitant care permitted or prohibited during the trial {11d}

There are no restrictions to concomitant therapy beyond exclusion based on current treatment with drugs that could increase ivermectin exposure.

### Provisions for post-trial care {30}

The study will contract a sponsor liability insurance coverage by ISGlobal, which is in line with applicable laws and/or regulations.

### Outcomes {12}

Primary:

#### Efficacy

Infection incidence in the most vulnerable age group (children under 5 years of age) for 6 months from the moment of the first MDA round in their community.

Infection incidence has been chosen as the primary endpoint based on the WHOs PPC for endectocides, which states that in areas of moderate to high transmission, the minimally acceptable efficacy criterion would be “at least 20% reduction in the incidence of clinical malaria (as primary outcome) and incidence of infection (as secondary outcome) in children under 5, lasting for at least 1 month following a single regimen.” The monthly frequency of visits has been chosen given that infected children will receive treatment with Coartem (artemether-lumefantrine), which has an established post-treatment prophylaxis period of 2 weeks [50, 51], monthly visits will allow for at least 14 days at-risk between visits.

Infection incidence will be determined in a cohort of children in the target age that is followed prospectively for 6 months post-receipt of the first dose in the community by a dedicated field team. Each child will be tested at home using RDTs (Parasite Lactate Dehydrogenase (pLDH) and HRP2 based). Two different tests will be used given the known persistence of HRP2 antigen after treatment with up to 50% residual positivity after 6 weeks while LDH-based tests become negative in just 48 h [52]. Quality control of RDT results will include PCR assessment of 10% of the samples.

#### Safety

Rate of AEs and serious adverse events (SAEs) and the difference between ivermectin and albendazole.

Secondary

1. Complementary efficacy analysis methods

- T﻿ime to first positive RDT in children in the cohort
- Molecular force of infection in a subset of children in the cohort
- Malaria case incidence in all ages presenting at a health facility
- Malaria prevalence in all ages one month after the last dose
- Multiplicity of infection in all ages one month after the last dose

2. Complementary safety variables

- Observed tolerability of the dose
- Rate of SUSARs
- AEs and SAEs by organ system

3. Pharmacokinetics

- Whole blood concentrations of ivermectin in dried blood spots.

4. Impact of ivermectin against selected ectoparasites and NTDs in the dosage regimen chosen

- Serial prevalence of scabies using a simplified version of the algorithm described by Mahe et al. [53] which consists of two questions and an evaluation of exposed skin on hands, knees, elbows, armpits, wrists, and feet.
- Serial prevalence of head lice by visual inspection over three minutes in scalp sites of predilection for head lice: the back of the ears, temples, and neck [54].
- Serial prevalence of Tunga penetrans via visual inspection of the skin in both feet and applying the Fortaleza scale [55].
- Serial prevalence and severity of bed bug infestation by direct questions about bedbugs in the house, and visual inspection of exposed skin and sleeping rooms for evidence of bed bug bites or bed bug infestation.

5. Prevalence - incidence correlation

- Malaria infection incidence in children (under 5 years) in the pediatric active cohort at community and health facility levels
- Malaria prevalence at all ages

6. Relationship between active and passive surveillance

- Infection incidence in children at the community level (active surveillance)
- Infection incidence in children at the health facility level (passive surveillance)

7. Performance of RDTs vs PCR

- Results correlation between HRP2 and pLDH-based RDTs
- Results correlation between RDTs and PCR
- Proportion of RDT-negative but PCR-positive infections

## Participant timeline {13}

**Table:** 

Procedures in treatment/safety cohort	Census and clustering Months −10 to −4	Treatment enrollment visit 1–30 days before dosing	Study visit 1(S) Day 1	Study visit 2(PK) Day 2	Study visit 3(S) Day 3 +/−1 day	Study visit 4(S) Day 6 +/− 1 day	Study visit 5(S) Day 31 +/−3 days	Study visit 7(S) Day 36 +/−1 day	Study visit 8(S) Day 61 +/-3 days	Study visit 10(S) Day 66 +/−1 day	Final safety visit 11(S) Day 91 +/−1 day	End of passive safety surveillance Day 365+/−3 days	Final pregnancy safety visit(S) ^d^ Day 371 +/−3 day
Informed consent census *(separate protocol)*	X												
Demographic data collection	X												
Cluster randomization	X												
Informed consent for treatment		X					X^e^		X^e^				
Complete visit template		X	X	X	X	X	X	X	X	X	X		X
Anthropometry		X					X		X				
Assessment of selected NTDs ^a^		X^a^					X^a^		X^a^		X^a^		X^a^
COVID response^a^		X					X		X		X		X
Review of infant vaccination card (if applicable)		X					X		X		X		
Collection of pharmacology samples ^a^			X	X	X								
Collection of parasite response samples ^b^			X				X		X				
Urine pregnancy test ^c^			X				X		X		X		
Administer study intervention			X				X		X				
Review of side effect personal diary						X		X		X			
Passive surveillance of side effects				X------------------------------------------------------------------------------X									
Adverse event review and evaluation				X-------------------------------------------------------------------------------------------------X									
Active pregnancy surveillance							X		X		X	X	X
Passive pregnancy surveillance (health facility)							X--------------------------------------------------------------------------------X						

^a^Randomly selected sub-sample

^b^Randomly selected sub-sample of children under 5 that also receive treatment (>15 kg)

^c^In participating female participants aged 13-49

^d^Only pregnancies occurring post-treatment

^e^Only subjects not previously enrolled

**Table:** 

Procedures in efficacy (active pediatric cohort)	Census and clustering Months −10 to −4	Enrollment/baseline(E) Visit 1, day 1	Study visit 2(E) Day 31 +/−3 days	Study visit 3(E) Day 61 +/− 3 days	Study visit 4(E) Day 91 +/−3 days	Study visit 5(E) Day 121 +/−3 days	Study visit 6(E) Day 151 +/−3 days	Study visit 7(E) Day 181 +/−3 days
Informed consent for census	X							
Demographic data collection	X							
Randomization	X							
Informed consent		X						
Anthropometry		X			X			X
Review infant vaccination card		X			X			X
Complete visit template		X	X	X	X	X	X	X
Malaria RDT		X	X	X	X	X	X	X
Malaria treatment ^a^		X	X	X	X	X	X	X
Assessment of selected NTDs		X	X	X	X	X	X	X
COVID response^b^		X	X	X	X	X	X	X
Malaria parasite immune response samples ^b^		X	X	X	X	X	X	X
*Plasmodium* PCR for quality control and molFOI^c^		X	X	X	X	X	X	X
Passive incidence surveillance at health facilities (all ages)		X-------------------------------------------------------X						

^a^Only if RDT is positive (see case definition)

^b^Only in a randomly selected sub-sample

^c^Only in a randomly selected 10% sub-sample

### Sample size {14}

The sample size is based on the main outcome (malaria incidence in a cohort of children followed prospectively in the cluster’s core area for six months), an outcome chosen based on WHO’s PPC for endectocides. This sample size has been corrected for cluster effect following Hayes and Bennett formula for unmatched rates [56].

The magnitude (20% reduction) was selected based on WHO’s recommended PPC for endectocides [57]. Additionally, the results of the RIMDAMAL trial suggest that a 20% reduction in malaria incidence can be achieved using a dosing regimen with a similar cumulative dose [40], these results although not statistically significant do point towards an expected effect size.

The final sample size is dependent on the population distribution as identified in the demography protocol.

The number of children per cluster can vary in order to streamline the cluster generation once the demographic data is available. The power, significance level, and target effect size will remain unchanged. The final number of clusters depends on the cluster size (the number of children in the active pediatric cohort recruited in the core of all clusters).

The target number of children to be included in every cluster is defined by the expected enrollment rate and loss to follow-up rate. We have estimated these as 15% and 20% respectively. So, for a cluster size to be 24 i.e., 24 children in the cohort finishing the study, the cluster must include 35 eligible children in the beginning (35 eligible of which an estimated 5 [15%] do not consent, 30 consent, of which 6 [20%] are LFU)

The number of clusters required increases exponentially as the number of children per cluster (cluster size) decreases. This is particularly marked below 13 children per cluster. The number of children presented is the number of eligible children required at the beginning, not accounting for 15% non-consenting and up to 20% LFU.

Peak incidence age: < 5 years

Power: 80%

Significance level: 5%

Expected incidence in the control group (λ0): 4.20

Expected incidence in the intervention (λ1): 3.36

Incidence reduction: 20%

Optimal cluster size: 20 children (assuming 3 non-consenting and 3 LFU)

Follow-up time: 6 months starting post-community dosing

Coefficient of variation (km): 0.35

People treated per cluster: approximately 300

Optimal total clusters required: 159 clusters (53 per arm)

The final sample size may have to be adjusted as by the table below. However, the power and effect size will remain constant as does the ceiling of treatable adults (not to exceed 48,000 eligible adults).

**Table:** 

Total clusters needed	Total children eligible per cluster	Non-consenting 15%	Included	LFU 20%	Final size	Total children in clusters	Total children enrolled	Total children completing trial
147	35	5	30	6	24	5145	4373	3499
149	30	5	26	5	20	4470	3800	3040
153	25	4	21	4	17	3825	3251	2601
159	20	3	17	3	14	3180	2703	2162
174	15	2	13	3	10	2610	2219	1775
195	10	2	9	2	7	1950	1658	1326
291	5	1	4	1	3	1455	1237	989

#### Preliminary data

The baseline incidence and the coefficient of variation used for the sample size calculation for Mozambique are considered robust as they have been obtained from a recently finished cluster-randomized trial in Mopeia, the very same district where BOHEMIA will be conducted. The previous trial assessed the cost-effectiveness of combining LLIN and IRS [58].

#### Calculations

Based on the demographic data to be confirmed in the preceding census, the clusters are planned to include approximately 420 people, of which 280 will be above 4–5 years of age and potentially eligible for treatment and 140 will be children under 5 potentially eligible for follow-up. There will be 53 clusters per arm in Mozambique.

Clusters in Mozambique will be randomized in three arms, resulting in inclusion of approximately 45,000 participants to receive treatment or control and approximately 2000–4000 children to be followed for 6 months.

With this sample size, the study has 80% power at 5% significance to detect a 20% reduction in malaria incidence infection, from 4.20 cases/child-year at risk to 3.36 for the human-only intervention, using a coefficient of variation (km) of 0.35 for Mopeia/Mozambique. This value for km has been back-calculated from a cluster randomized trial recently conducted in the same study site [58].

For the human and livestock intervention, this sample size has 80% power to detect a further 20.5% reduction in malaria incidence infection between the human and human + livestock intervention arms, from 3.36 cases/child-year at risk (the lower incidence expected in the human only arm) to 2.67.

The total number of clusters needed per arm has been increased by one for all scenarios to account for potential LFU of a whole cluster (withdrawal of community consent, displacement, etc.)

### Recruitment {15}

Community mobilization teams will visit all villages and meet with leaders and community members to inform them of the upcoming trials. The information methods will include door-to-door visits and focal group discussions (see stand-alone community mobilization plan)

## Assignment of interventions: allocation

### Sequence generation {16a}

Once all clusters in the district are defined, the target sample will be randomized using a computer-generated algorithm. Clusters will be randomized 1:1:1 into three study arms. The treatment status of each cluster will be assigned during cluster delineation and then transmitted to the population during a randomization ceremony

### Concealment mechanism {16b}

The sequence will be encrypted and the password kept by an independent data manager

### Implementation {16c}

The sequence was generated by an independent data manager. Recruitment is carried on by unblinded field workers

## Assignment of interventions: blinding

### Who will be blinded {17a}

The study will be open-label but outcome assessor-blinded participants in the ivermectin and control arms will receive products that differ in aspect and numbers. Participants will be informed that they are taking one of two anthelminthics. The sponsor and the study statistician will remain blinded to the assignment of each cluster group. All unblinded DSMB reports will be prepared by an independent statistician

### Procedure for unblinding if needed {17b}

The sponsor personnel will analyze data semi-blinded (groups 1–2–3) and only unblind after completion.

## Data collection and management

### Plans for assessment and collection of outcomes {18a}

Risk-based monitoring of the trials shall be conducted by qualified and experienced CRAs and shall be in accordance with a Monitoring Plan that clearly describes the strategy, methods, responsibilities, and requirements for monitoring the trials.

In accordance with applicable regulations, GCP, and ISGlobal procedures, CRAs shall meet with the site prior to the start of the study to review with the site staff the protocol and their responsibilities to satisfy regulatory, ethical, and ISGlobal requirements. When reviewing data collection procedures, the discussion shall also include training on the electronic source requirements, entry into the clinical trials’ database, as well as access and security procedures.

The investigator and the head of the institution (where applicable) agrees to allow the monitor direct access to all relevant documents. The investigator must ensure the provision of reasonable time, space, and qualified personnel for monitoring visits.

As the trials make use of electronic source reporting, most data will be monitored remotely, and queries will be resolved electronically to reduce monitoring time on site.

Upon completion or premature discontinuation of the study, the CRA will conduct site closure activities with the investigator and site staff in accordance with applicable regulations, GCP, and ISGlobal SOPs.

### Plans to promote participant retention and complete follow-up {18b}

Data from withdrawn participants is to be included in the analysis outside explicit requirement. In cases of voluntary discontinuation, it should be established whether consent remains for sample storage. Should the consent be withdrawn, the stored sample will be destroyed and the withdrawal noted in the source notes and CRF. The site shall send a sample destruction letter to the laboratory for actioning. The laboratory shall return a copy of the completed destruction letter once the sample has been destroyed.

### Data management {19}

Data will be collected through a comprehensive electronic data capture (EDC) system with digital data entry forms deployed and stored on the broad OpenHDS (Health and Demographic System). Specifically, the study questionnaires will be designed and deployed on the Open Data Kit (ODK) platform running on the open-source Android operating system. Data transfer from mobile devices (tablets) to servers will be executed using ODK-Collect and Mirth. Data validation will take place both at (a) point of entry via restricted and confirmatory steps, and (b) on the server, via aggregate-level quality checks and alerts. Backups of both the raw input data and the processed data will be stored on the main project server as well as at each site. An audit trail/log of all data modifications, both in terms of inputs and edits, will be generated automatically and available to RAs or authorized study team members upon request.

Every effort will be made to ensure the protection of participant data. In general terms, this means that no identifying data will be transferred to any server unless both (a) its transfer falls within the scope of the project’s protocol, and (b) it is appropriately encrypted and secure.

Paper-based source (e.g., hospital records) will be scanned into the safety database locally and entered into the trials´ databases. Paper-based records will redact all personal identifiers of the participant (e.g.; name of the participant, address or telephone number, hospital file number) and will be stored securely at the site.

Clinical data management shall be performed in accordance with applicable ISGlobal standards and data cleaning procedures.

While completed eCRFs are reviewed by ISGlobal Clinical Research Associates both remotely and at the study site, omissions or inconsistencies detected by subsequent eCRF review may necessitate clarification or correction of omissions and inconsistencies with documentation and approval by the investigator or an appropriately qualified designee. In all cases, the investigator remains accountable for the study data.

The investigator will be provided with a physical data drive (in form of CD-ROM, USB, or HDD) of the final version of the data generated by the project once the database is archived and the study report is complete and approved by all parties. The physical data drive will be compliant with international requirements for data storage and retrieval.

### Confidentiality {27}

Every effort shall be made to protect participant privacy and confidentiality to the extent permitted by law. All trial-related information shall be stored securely at the trial site.

All paper-based participant information shall be stored in lockable file cabinets in areas with access limited to trial staff. Data collection, process, and administrative forms, laboratory specimens, and other reports shall be identified by a coded number only, to maintain participant confidentiality. Participant identifiers (PID) shall contain the number of the trial site and the individual participant number allocated at randomization.

All records that contain names or other personal identifiers, such as locator forms, participant identification code list and informed consent forms, shall be stored separately from study records identified by code number. Only the trial health-care personnel directly responsible for the participant’s care and one designated data management member of staff shall have access to these records.

All databases shall be secured with password-protected access systems.

If the participant’s name appears on any other document (e.g., pathologist report), it shall be obliterated before a copy of the document is supplied to ISGlobal.

Participants’ study data, as identified by PID number only, shall not be released without the participant’s written permission, except as necessary for review and monitoring by:

•Authorized study representatives

•Local and international RAs

•IECs/ IRBs

•Sponsor Monitors

•Sponsor and third-party Auditors

When the results of the study are published, the participant’s identity shall remain confidential

### Plans for collection, laboratory evaluation and storage of biological specimens for genetic or molecular analysis in this trial/future use {33}

Blood samples will be stored in the clinical trial sites in silica gel as a desiccant in −20°C freezers and/or 4°C fridges according to the specific requirements of the processing lab. Samples to be sent to Basel Hospital (Switzerland), KEMRI (Kenya) and ISGlobal (Spain) will be stored separately in different bags, each identified with the corresponding identification number.

Samples may be stored by ISGlobal or its contractor/s for a period of 5 years. The samples will be stored in an accredited, secure, access-controlled laboratory. The samples will be identified by a participant ID and visit code until the end of the trials where after which they will be delinked so that they cannot be linked to a specific participant. Additional research regarding the use of ivermectin to control malaria may be conducted to evaluate hypotheses generated during data analysis, i.e., if a direct effect of ivermectin on the parasite is suspected based on epidemiological results. This includes parasite genetics, serological and immune response to the malaria parasite, and NTDs. Additional uses could include re-analyses with methods being developed during the course of the study, and to answer follow-up questions posed by stakeholders.

The positive RDTs will not be discarded. They will be stored at −20° for 5 years for the conduct of malaria transmission studies (parasite genetics) relevant to the study site (Mopeia). These studies are not part of the BOHEMIA clinical trial and will have independent protocols and approvals for their conduct

## Statistical methods

### Statistical methods for primary and secondary outcomes {20a}

Baseline characteristics in the intervention groups will be compared and assessed for similarity.

The primary outcome of these trials should be the incidence across 6 months of follow-up.

This study comprises three data collection strategies: at the community level with a pediatric active cohort and cross-sectional surveys, and at the health facility level with a passive surveillance system. The main analysis will be conducted at the community level and some secondary and exploratory outcomes will be assessed at the health facility level.

#### Analysis variables

Key analysis variables consist of baseline socio-demographic characteristics, malaria infection incidence (efficacy), and AEs and SAEs data (safety).

##### Efficacy analysis

The primary analysis will be the differential malaria incidence after the 6-month follow-up period between the intervention and control groups. Incidence will be based on monthly RDT results from the active cohort. Each RDT performed will be taken to represent one visit, which is equivalent to one child-month at risk. In the per-protocol correction, 14 days of time at risk for each treatment received (based on the post treatment prophylaxis provided by AL).

Differential incidence between intervention and control clusters will be assessed using a count data regression model accounting for the cluster design effect. Primary analysis will be assessed using generalized estimating equations.

Analysis in the multivariate model will be adjusted for potential confounders identified in the univariate analysis. Based on previous vector-control trials, the variables to be considered can be classified in the following categories: demographics (age and gender), socio-economics (head of household formal education, head of household occupied as a subsistence farmer, electricity in the household, livestock-human ratio, number of nets owned and distance to the nearest health facility), clinical (having experienced fever in the previous 48h, having slept under a bed net the previous night and infection of a sibling in the same household). A step-wise approach will be applied for building the final multivariate model, considering those variables with p-values lower than 0.20 in the univariate analysis.

To assess the bias due to LTFU, sensitivity analyses limited to participants who receive all treatments may be performed. Additional support will be obtained from a comparison of the baseline characteristics of individuals who remain vs. those that are LTFU.

Assessment of the direct benefit to the population that receives treatment will be done through passive surveillance at health facilities and cross-sectional studies to allow for a better risk-benefit analysis in this population.

##### Safety analysis

For the safety co-primary objective, AEs will be analyzed in total and by body system. Tables will show the number of AEs observed and the number and percentage of participants experiencing them within a system organ class or within preferred term category by severity or by relationship to the study product. Participants with multiple AEs within a category will be counted once under the maximum severity or the strongest recorded causal relationship to the study product. Differences between AE rates in the intervention groups will be assessed by means of generalized estimating equations to account for the cluster design.

A listing of SAEs reported to the DSMB will provide details of the events including severity, relationship to the study product, time between onset and last treatment, and the number of treatments administered and received by the study participant.

The number and percentage of study participants who discontinue treatment and who terminate from the study early will be tabulated by reason and treatment arm.

### Interim analyses {21b}

There will be three rounds of MDA separated by a month each. Safety data will be collected in the month following each administration. Given the delay in reporting and the ample experience with ivermectin in MDA, there will be no interim analyses

### Methods for additional analyses (e.g., subgroup analyses) {20b}

These are detailed in the SAP.

### Methods in analysis to handle protocol non-adherence and any statistical methods to handle missing data {20c}

Missing data frequency and patterns will be studied for the main variables and, if appropriate, multiple imputation techniques will be considered. Unused and spurious data will be excluded from data analysis as soon as it is recognized

Any deviations from the statistical methods explained in the protocol will be described and justified in the final report.

The SAP will be reviewed by the DSMB and updated where necessary as a result of interim analyses of the data. A formal record will be kept detailing when the SAP was finalized. It will suffice to update the statistical analysis plan with the considerations suggested from the reviews as well as any protocol amendment.

A detailed review of the SAP will also be carried out prior to the study close-out to ensure any protocol amendments have been incorporated. Statistical changes such as rationale for newer or improved analytic methods for the data will be documented

### Plans to give access to the full protocol, participant level-data and statistical code {31c}

Per the contractual agreement, the database will be made publicly available upon completion of the project

## Oversight and monitoring

### Composition of the coordinating center and trial steering committee {5d}

ISGlobal is the coordinating center. The PI, CSO, project manager, GCP-QA focal person, and program officers work on a day-to-day basis for the management of the trials. There are also regular weekly meetings with other personnel including the financial officer and communications.

There is a Trial Steering Committee that meets twice a year and is composed of field experts and country representatives.

### Composition of the data monitoring committee, its role and reporting structure {21a}

An independent DSMB with seven members including representatives of each study country has been assembled for this study. The details for the operation and responsibilities of the DSMB are defined in a DSMB Charter. The Charter delineates the composition, duties, responsibilities, and procedures of the DSMB, the data required at each meeting as well as any analyses that will be conducted.

After each DSMB meeting, the Chairperson will issue a written report describing all recommendations

### Adverse event reporting and harms {22}

Participants will be asked to record signs and symptoms associated with known ADRs in the diary for review by the field worker during the post-dosing safety visit occurring 1 month post dose.

The participant will be asked to provide information on any other events, physician visits or hospitalizations, and treatment taken during day 0 through day 6 during the post dosing safety visit.

Participants will be asked about any AEs occurring after the 6th day and up to the next dosing day. As the IP will no longer be present in the participant’s system at detectable levels, these events will be deemed unrelated to the IP. These events will be recorded on adverse event logs, which form part of the source data and will not be included in the data analysis. These unrelated events will be reported to IRBs and DSMB in 6 monthly line listings unless local regulations specify otherwise.

Participants shall be encouraged to contact the Investigator by telephone to report severe events in case the investigator deems an ad hoc visit by the participant to the site or local clinic is required.

The investigator and/or sub-investigator with assistance from delegated study staff will visit local hospitals in the district to review in- and out-patient records to ensure all adverse events for trial participants are captured. The investigator or appropriately qualified sub-investigator shall determine the severity and causality for all events.

All AEs and SAEs occurring from the time a volunteer consents to participate in the study until 4 weeks after he or she has completed or discontinued the IP must be recorded in the Patient’s electronic records and trial database. BOHEMIA will use MedDRA coding and CIOMS forms to ensure standardization of terminology across the trial.

Importantly, SAEs will have to be reported, either by email or by Fax, by the site PI/Co-PI or sub-investigator to IntuVigilance Limited within 24 h of awareness of an SAE.

### Frequency and plans for auditing trial conduct {23}

To ensure compliance with GCP, all applicable regulatory requirements, as well as the protocol, ISGlobal, Unitaid (the funder), RAs, and/or ethics committees may conduct a routine quality assurance audit or regulatory inspection of this study. Additionally, in instances where there is a high occurrence of non-compliances at a trial site or misconduct is suspected, any one of the parties may institute a for-cause audit/inspection.

Such audits/inspections can occur at any time during or after the completion of the study. If an audit or inspection occurs, the investigator and the research institution agree to allow the auditor/inspector direct access to all relevant documents and to allocate his/her time and the time of his/her staff to discuss findings and any relevant issues.

### Plans for communicating important protocol amendments to relevant parties (e.g., trial participants, ethical committees) {25}

These trials shall be conducted under the auspices of properly constituted Ethics Committees as defined by ICH-GCP E6 (R2) Guidelines. These committees shall review and approve all aspects of the study and subsequent amendments as stipulated by the ICH-GCP Guidelines prior to and during the conduct of the study.

### Dissemination plans {31a}

A publication policy and publication have been completed and signed off by all consortium members. Dissemination of study products in conferences or publications will be conducted following international guidelines. Findings from this study will not be disseminated without adequate leadership and representation of local researchers.

## Discussion

Implementing such a large trial under GCP-ICH standards in remote rural settings in Mozambique is challenging at several levels:

1. ICH standards have been developed primarily for hospital-based work in high-income countries and they do not always take into consideration the environmental or cultural context of rural Africa. A clear example is the legal guardianship of minors. Can the primary caretaker (an “auntie”) provide consent for a child if both parents are absent or dead even if this primary caretaker does not have written proof of legal guardianship?
2. There is a need to have community buy-in by involving local personnel in the field work, yet the scarcity of trained personnel increases the costs and length of training
3. Cluster randomization brings additional complexities as community engagement and early handling of rumours requires a fully dedicated team
4. Malaria is a seasonal disease, and implementing a mass drug administration program during the rainy season carries enormous logistical challenges including degraded roads, floodings, and general supply constringent
5. The Mozambican team faced five cyclones and severe floodings followed by a cholera outbreak. This means a surge in the number of deaths in study participants. This put a serious strain on the local team in order to keep the strict reporting timelines required by ICH. Appropriate resources were put in place including the procurement of satellite phones for usage in areas with no cell phone network
6. Collecting large amounts of data from the field with limited connectivity requires complex logistical operations such as physically transporting tablets to an area with 3G coverage for upload and refresh

## Trial status

Protocol version 4.2 dated 13 March 2022. Recruitment started on 15 March 2022 in Mozambique. Recruitment was completed in July 2022. Follow-up of the active pediatric cohort will continue until October 2022. Follow-up of the pregnancy cohort will continue until March 2023

We submit the protocol after finishing recruitment but before the completion of the follow-up of the last patient envisioned for March 2023. This is mentioned in the structured summary and is because of the following reasons:

- Delayed ethical approval given discussions between the WHO ethics committee and that of Mozambique which were only solved the day before implementation.
- Implementation difficulties such as floodings, a cholera outbreak and rumors for the first 4 months required the full attention of the study team.
- The Tanzanian government issued a stop work order for the district of Rufiji given the potential this meant that the team had to seek an alternative site while implementing in Mozambique. This has been found in Kenya and the protocol is currently undergoing review there.

## Data Availability

Per the contractual agreement, the database will be made publicly available upon completion of the project

## Abbreviations

ADR: Adverse drug reaction
AE: Adverse event
AL: Artemether-lumefantrine
AUC: Area under the curve
CDC: Centers for Disease Control and Prevention
CIBS: Ethics Committee of CISM
CISM: Centro de Investigação em Saúde De Manhiça
CNS: Central nervous system
CRA: Clinical Research Associate
CRF: Case Report Form
DEC: Diethylcarbamazine
DOT: Directly observed treatment
DSMB: Data and Safety Monitoring Board
eCRF: Electronic Case Report Form
EDC: Electronic data capture
EMA: European Medicines Agency
EU: European Union
FDA: *Food and Drug Administration*
GCP: *Good Clinical Practice*
GSK: *GlaxoSmithKline*
HFW: Health facility workers
ICH: *International Conference on Harmonisation*
IHI: Ifakara Health Institute
IP: *Investigational Product*
IRB: *Institutional Review Board*
IRS: Indoor residual spraying
LF: Lymphatic filariasis
LFU: Lost to follow-up
LLIN: Long-lasting insecticidal nets
LMP: Last menstrual period
MDA: Mass drug administration
MoH: Ministry of Health
MUAC: Mid-upper arm circumference
NDA: New drug application
NMCP: National Malaria Control Programme
NTD: Neglected tropical disease
PCR: Polymerase chain reaction
PID: Participant identifiers
PI: Principal Investigator
PK: Pharmacokinetics
pLDH: Parasite lactate dehydrogenase
PMI: President’s *Malaria* Initiative
PPC: Preferred product characteristics
RA: Regulatory authority
RDT: Rapid diagnostic test
REC: Research Ethics Committee
SAE: Serious adverse event
SAP: Statistical analysis plan
SMC: Seasonal malaria chemoprevention
STHs: Soil-transmitted helminths
SUSAR: Suspected unexpected serious adverse reaction
VCAG: Vector Control Advisory Group
WHO: World Health Organization
